# Clara Cell 10-kDa Protein Gene Transfection Inhibits NF-κB Activity in Airway Epithelial Cells

**DOI:** 10.1371/journal.pone.0035960

**Published:** 2012-04-25

**Authors:** Xiao-Bo Long, Shuang Hu, Nan Wang, Hong-Tao Zhen, Yong-Hua Cui, Zheng Liu

**Affiliations:** Department of Otolaryngology-Head and Neck Surgery, Tongji Hospital, Tongji Medical College, Huazhong University of Science and Technology, Wuhan, People's Republic of China; French National Centre for Scientific Research, France

## Abstract

**Background:**

Clara cell 10-kDa protein (CC10) is a multifunctional protein with anti-inflammatory and immunomodulatory effects. Induction of CC10 expression by gene transfection may possess potential therapeutic effect. Nuclear factor κB (NF-κB) plays a key role in the inflammatory processes of airway diseases.

**Method/Results:**

To investigate potential therapeutic effect of CC10 gene transfection in controlling airway inflammation and the underlying intracellular mechanisms, in this study, we constructed CC10 plasmid and transfected it into bronchial epithelial cell line BEAS-2B cells and CC10 knockout mice. In BEAS-2B cells, CC10's effect on interleukin (IL)-1β induced IL-8 expression was explored by means of RT-PCR and ELISA and its effect on NF-κB classical signaling pathway was studied by luciferase reporter, western blot, and immunoprecipitation assay. The effect of endogenous CC10 on IL-1β evoked IL-8 expression was studied by means of nasal explant culture. In mice, CC10's effect on IL-1β induced IL-8 and nuclear p65 expression was examined by immunohistochemistry. First, we found that the CC10 gene transfer could inhibit IL-1β induced IL-8 expression in BEAS-2B cells. Furthermore, we found that CC10 repressed IL-1β induced NF-κB activation by inhibiting the phosphorylation of IκB-α but not IκB kinase-α/β in BEAS-2B cells. Nevertheless, we did not observe a direct interaction between CC10 and p65 subunit in BEAS-2B cells. In nasal explant culture, we found that IL-1β induced IL-8 expression was inversely correlated with CC10 levels in human sinonasal mucosa. *In vivo* study revealed that CC10 gene transfer could attenuate the increase of IL-8 and nuclear p65 staining in nasal epithelial cells in CC10 knockout mice evoked by IL-1β administration.

**Conclusion:**

These results indicate that CC10 gene transfer may inhibit airway inflammation through suppressing the activation of NF-κB, which may provide us a new consideration in the therapy of airway inflammation.

## Introduction

Clara cell 10-kDa protein (CC10), also known as Clara cell secretory protein, uteroglobin, is a founding member of the newly recognized secretoglobin superfamily. It is constitutively expressed by the mucosal epithelial cells lining all organs that encounter the outer environment, including lung and nose [1]. CC10 possesses anti-inflammatory and immunomodulatory effects. Compared with wild-type mice, CC10 knockout mice demonstrate exaggerated airway inflammation provoked by allergic responses and bacterial and viral infection [2]. Reduced levels of CC10 have been correlated with allergic and inflammatory airway diseases, including asthma, allergic rhinitis, and sinusitis [3], [4], [5].

Airway epithelial cells provide a complex barrier for innate host defense. They can sense the external stimuli, such as invading pathogens and allergen exposure, and connect the innate and adaptive immunity [6], [7], [8]. When triggered by airborne threats, airway epithelial cells are capable of producing a variety of cytokines and chemokines such as interleukin (IL)-8, RANTES, and granulocyte-macrophage colony-stimulating factor, and lead to subsequent inflammation [9], [10]. IL-8 is first isolated from monocytes and acts as a neutrophil attractant [11], which is generally accepted as an important mediator in airway inflammation. Previous studies have revealed that neutrophils and IL-8 are associated with severe asthma and the exacerbation of acute asthma induced by human rhinovirus [12]–[15]. Compared with controls, the elevated levels of IL-8 have also be detected in the nasal discharge and sinus mucosa of chronic rhinosinusitis patients [16], [17], underscoring an important role of IL-8 in the upper and lower airway diseases. Of the many signaling cascades activated in airway epithelium in response to stimuli, nuclear factor κB (NF-κB) has been considered as one of the most important for the regulation of inflammation [18]. The NF-κB pathway impacts a number of key biological processes and regulates the transcription of many proinflammatory genes relevant to allergic and inflammatory airway diseases, such as IL-8, eotaxin, and cyclooxygenase-2, etc [19]. On the other hand, NF-κB can be activated in response to cytokines, mitogens, physical and oxidative stress, and microbial products [20]. For example, a classical response in airway inflammation is that IL-1β activates NF-κB pathway and then induces the expression of IL-8 in airway epithelial cells [21].

Given the anti-inflammatory function of CC10, in this study, we explored whether induction of CC10 protein expression through gene transfection can suppress IL-1β induced IL-8 production in airway epithelial cells and whether this effect is mediated through inhibiting NF-κB signaling pathway.

## Materials and Methods

### Subjects and ethic statement

Discarded human inferior turbinate mucosa from two patients undergoing partial inferior turbinectomy because of inferior turbinate hypertrophy were used as positive controls of CC10 expression in western blot analysis. To study the effect of CC10 on IL-1β induced IL-8 production in airway mucosa, 10 chronic rhinosinusitis (CRS) patients (6 male and 4 female, age range, 24–46 years) and 10 control subjects (7 male and 3 female, age range, 19–42 years) including 5 patients with mucus retention cyst of maxillary sinus, 2 patients with mucocele of maxillary sinus, and 3 patients with maxillofacial trauma that underwent endoscopic sinus surgery were recruited. Control subjects did not show obvious anterior ethmoid inflammation on coronal CT scanning and endoscopy and none had a history of persistent mucopurulent drainage. Diseased sinus mucosal tissues from most hypertrophied and hyperemic regions were collected from CRS patients and macroscopically normal anterior ethmoid mucosal tissues from controls were obtained during surgery. This study was approved by the ethical committee of Tongji Medical College of Huazhong University of Science and Technology (Permit Number: 20051007).

All mice were used following protocols approved by the Animal Care and Use Committee of Tongji Medical College of Huazhong University of Science and Technology (Permit Number: 20051007).

### Cell culture

The adenovirus 12 SV40-transformed human bronchial epithelial cell line, BEAS-2B cell, was purchased from American Type Culture Collection (Manassas, VA, USA). The BEAS-2B cells were cultured in DMEM/F12 (Gibco, Carlsbad, CA, USA) supplemented with 5% heat-inactivated fetal bovine serum, 2 mM L-glutamine, 100 U/mL penicillin, and 100 µg/mL streptomycin (Gibco). The cells were incubated at 37°C with 5% carbon dioxide in humidified air.

### CC10 plasmid construction and cell transfection

The human CC10 coding region gene, including a 276-bp sequence, was amplified from homogenized human inferior turbinate mucosa by RT-PCR. The sequences of PCR primers for CC10 used in this study were as follows: sense, 5′-AGAGACAGGCCAGAGCATCC-3′; antisense, 5′-GCAGAGGCTGGAGCAGTTG-3′. The PCR products were cloned into a TA expression vector (Invitrogen, Carlsbad, CA, USA), and the sequence of the CC10 coding region was confirmed by sequencing [22]. The resulting plasmids (pCC10) were propagated in Escherichia coli, purified through cesium chloride gradient, and confirmed to be free of endotoxin [22]. For the real-time PCR, ELISA, and western blot experiments, the BEAS-2B cells were seeded in 6-well plates at a concentration of 5×10^5^ cells per well. BEAS-2B cells approaching 80–90% confluence were transfected with 4 µg pCC10 with 10 µL lipofectamine 2000 (Invitrogen) following manufacturer's protocol. Twenty-four hours later, the medium was changed to a serum-free one. Cells were cultured for another 24 hours and then were stimulated with or without 10 ng/mL IL-1β (R&D Systems, Abington, UK). The cells transfected with empty plasmid pcDNA3.1 (mock) were used as negative controls for CC10 gene transfection.

### Quantitative real-time PCR assay of IL-8

To detect IL-8 mRNA expression level, six hours after IL-1β treatment, BEAS-2B cells were harvested and total RNA was extracted by using Trizol (Invitrogen) and treated by using a DNA-free kit (Ambion, Austin, TX, USA) to remove contaminating DNA. cDNA was reverse transcribed from 1 µg of total RNA with random hexamer primers using M-MLV RTase cDNA synthesis kit (Takara Biotechnology, Dalian, China). Quantitative PCR assay of IL-8 was performed by using the SYBR Premix Ex Taq kit (Takara Biotechnology) with the specific primers on Fast Real-Time PCR 7700 System (Applied Biosystems, Foster City, CA, USA) as previously described [5]. Amplification was as follows: 95°C for 1 minute, followed by 40 cycles of 95°C for 15 seconds, 60°C for 30 seconds and 72°C for 30 seconds. GAPDH was used as a housekeeping gene for normalization and ‘no template’ sample was used as a negative control. The primers used were as follows: IL-8 forward primer, 5′-ACTTTCAGAGACAGCAGAGCACACA-3′; IL-8 reverse primer, 5′-CCTTCACACAGAGCTGCAGAAATC-3′; GAPDH forward primer 5′-ACCCAGAAGACTGTGGATGG-3′; GAPDH reverse primer, 5′-TTCTAGACGGCAGGTCAGGT-3′. Relative gene expression was calculated by using the comparative CT method as described previously [5]. One cell condition without any treatment was employed as a calibrator.

### IL-8 ELISA

To detect the IL-8 protein levels, twenty-four hours after IL-1β treatment, the supernatants were harvested and stored at −80°C for analysis. Supernatants were tested for the amounts of IL-8 by using a commercial kit (R&D Systems) according to the manufacturer's recommendations. The lower detection limits of ELISA kit were 8 pg/mL.

### NF-κB luciferase reporter assay

For luciferase reporter assay, BEAS-2B cells were seeded in 12-well plates at a concentration of 1×10^5^ cells per well. BEAS-2B cells approaching 80–90% confluence were transiently transfected with either 1 µg of pCC10 or pcDNA3.1 mock plasmid along with 1 µg of pGL4.32[*luc2P*/NF-κB-RE/Hygro] vector (Promega, Madison, WI, USA) and 0.1 µg of pGL4.74[hRluc/TK] vector (Promega) by using lipofectamine 2000 (Invitrogen). The pGL4.32 plasmid is a NF-κB reporter vector. It contains NF-κB response elements and firefly luciferase gene. The pGL4.74 plasmid contains renilla luciferase gene and was used as a system control. Twenty-four hours after transfection, the medium was changed to a serum-free one. Cells were cultured for another 24 hours and then stimulated with 10 ng/mL IL-1β for 4 hours. Viability and cell recovery of BEAS-2B cells were similar in each group. Cell lysates were harvested and processed with the Dual-Luciferase Reporter Assay System (Promega). The firefly luciferase activity was normalized to renilla luciferase activity for each sample, and NF-κB activity was expressed as relative luciferase activity.

### Immunofluorescence of CC10 in BEAS-2B cells

To analyze the expression of transduced gene, the BEAS-2B cells were seeded in 6-well plates and transfected with pCC10 or mock for 24 h as mentioned above, and then 1×10^5^ pCC10 transfected BEAS-2B cells were seeded in 12-well plates pre-coated with sterile coverslips. Twenty-four hours later, the coverslips were fixed and incubated with a rabbit anti-human CC10 antibody (Santa Cruz Biotechnology, CA, USA) at a dilution of 1∶200 as previously described [23]. Control isotype rabbit IgG was used as a negative control. A FITC marked goat anti-rabbit antibody (1∶100; Santa Cruz) was used as a secondary antibody. The cell nucleus was stained with DAPI (Sigma, St. Louis, MO, USA).

### Western blot analysis

To detect the expression of transduced genes, total proteins were extracted from BEAS-2B cells 48 h after transfection. Proteins extracted from human inferior turbinate mucosa were used as a positive control for CC10 expression. To examine the effect of CC10 gene transfer on NF-κB activity, BEAS-2B cells were seeded in 6-well plates, transfected, and treated with IL-1β as mentioned above. Thirty or sixty minutes after IL-β treatment, total and nuclear proteins were extracted according to the method described previously [24]. The proteins were separated by 10% sodium dodecyl sulfate–polyacrylamide gel electrophoresis and then transferred to nitrocellulose membranes. After blockage of non-specific binding sites, the membranes were incubated with rabbit anti-human CC10 antibody (1∶100, Santa Cruz), or rabbit anti-human p65, phospho-IκB-α, or phospho-IκB kinase (IKK) α/β antibody (1∶500, Cell Signaling Technology, Danvers, MA, USA), or mouse anti-human β-actin antibody (1∶400, Boster, Wuhan, China) at 4°C for 24 h. The membranes were then washed and incubated with alkaline phosphatase conjugated goat anti-rabbit antibody or rabbit anti-mouse antibody (Jackson, West Grove, PA, USA) at 37°C for 1 h. The immunoblots were visualized by use of a 5-bromo-4-chloro-3-indolylphosphate/nitroblue tetrazolium alkaline phosphatase color development kit (Roche, Indianapolis, IN, USA). Species and subtype matched antibodies were used as negative controls. The protein expression levels were semi-quantified by measuring the gray scale normalized to β-actin using the IS1000 image analysis software (Alpha innotech, CA, USA).

### Coimmunoprecipitation

To detect whether there was a potential interaction between NF-κB p65/p50 subunits and CC10 protein, BEAS-2B cells transfected with pCC10 were stimulated with 10 ng/mL IL-1β for 4 hours. Immunoprecipitation and immunoblotting were performed by using ProFound Mammalian Co-Immunoprecipitation Kit (Pierce, Rockford, IL, USA). The total cell proteins were extracted as previously described [24]. The proteins were immunoprecipitated by rabbit anti-human p65 antibody (Cell Signaling Technology) and rabbit normal serum as a negative control. For coimmunoprecipitation experiments, western blot was performed using both rabbit anti-human CC10 (1∶100, Santa Cruz) and rabbit anti-human p65 antibody (1∶500, Cell Signaling Technology). Control isotype rabbit IgG was used as a negative control of primary antibodies and human inferior turbinate tissues were used as positive controls for CC10 and p65 expression.

### Nasal explant culture

To study the effect of endogenous CC10 on the modulation of airway inflammation, sinonasal mucosa samples from 10 CRS patients and 10 controls were subject to nasal explant culture as previously described [25]. Briefly, the mucosal tissues were sectioned into multiple samples of approximately 6 mm^3^ and sections of tissues were placed on 0.4 µm well inserts (Millipore Corp., Billerica, MA, USA) in 2 mL of tissue culture medium [25], [26]. The tissue was oriented with the epithelium being exposed to the air, forming an air–liquid interface to mimic the in vivo situation. The tissue was cultured at 37°C with 5% carbon dioxide in humidified air. Tissues were incubated with 10 ng/mL IL-1β for 24 hours, and then the CC10 levels in tissue homogenates and IL-8 levels in supernatants were determined with ELISA kits according to the manufacturer's protocols. CC10 ELISA kits were purchased from BioVendor Laboratory Medicine Inc. (Brno, Czech Republic) and IL-8 ELISA kits were purchased from R&D Systems.

### Animal experiments

Homozygous CC10-knockout mice on C57BL/6 background were obtained from an intercross of heterozygous CC10-knockout mice (kindly provided by Dr. A. B. Mukherjee, National Institutes of Health, Bethesda, MD) [27], and germline transmission of the mutant CC10 allele was identified by PCR as described [28]. Mouse CC10 gene was constructed as previously described [15]. For mucosal gene transfer, mice received intranasal injection of 50 µL pcDNA3.1+ lipofectamine (mock; 10 µg plasmid/25 µL PBS +25 µL lipofectamine) or pCC10+ lipofectamine (pCC10; 10 µg plasmid/25 µL PBS +25 µL of lipofectamine) as previously reported [2]. Three days later [2], 20 µg IL-1β in 40 µL of PBS was administered intranasally (20 µL per nostril). Twenty-four hours after that, mice were sacrificed with sodium pentobarbital and sinonasal cavity structure was dissected, decalcified, embedded in paraffin, and sectioned as mentioned previously [29]. Immunohistochemical staining was conducted using the streptavidin–peroxidase complex method as described previously [5]. Rabbit anti-mouse IL-8 (1∶200, Abbiotec, San Diego, CA, USA) and rabbit anti-mouse p65 (1∶300, Cell Signaling) antibodies were used as primary antibodies. Color development was achieved with 3′, 3′-diaminobenzidine, which rendered positive cells brown. Control isotype rabbit IgG was used as a negative control. For quantitative analysis of the IL-8 staining in epithelium of nose mucosa, the mean optical density, which represents the integrated optical density divided by epithelium area, was measured by the Image-Pro Plus (Media Cybernetics, MD, USA) in 10 random selected high power fields. The number of nuclear p65 positive cells per millimeter of epithelium was counted as previously described [2]. Three days after transfection, the induction of CC10 expression in sinonasal epithelium was confirmed by immunohistochemical staining as previously described [2].

### Statistical analyses

Data are presented as mean±SD. All data were confirmed with a normal distribution and assessed for significance by paired or unpaired t-test or ANOVA as appropriate. Partial correlations were computed to describe the association between IL-8 protein and CC10 protein expressions in sinus mucosa tissues after incubate with IL-1β while adjusting for the influence of diagnostic groups. Differences were considered statistically significant at a *P* value less than 0.05.

## Results

### Induction of CC10 protein expression in pCC10 transfected BEAS-2B cells

**Figure 1:**
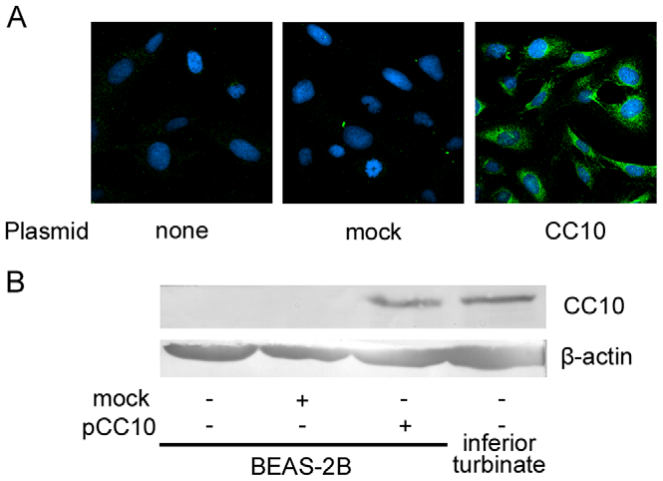
CC10 is overexpressed in pCC10 transfected BEAS-2B cells. CC10 expression was detected by means of (A) immunofluorescence staining and (B) western blotting. CC10 was mainly expressed in the cytoplasm of pCC10 transfected BEAS-2B cells. One representative experiment of the three independent experiments is shown. CC10, Clara cell 10-kDa protein. Proteins extracted from human inferior turbinate mucosa from 2 patients undergoing partial inferior turbinectomy were used as positive controls for CC10 expression.

First, we detected CC10 gene expression in the BEAS-2B cell line. No CC10 mRNA expression was found in BEAS-2B cells before or after IL-1β stimulation (data not shown). Neither was CC10 protein production found in BEAS-2B cells as detected by immunofluorescence staining or western blot analysis (Figure 1). Since our previous studies demonstrated the expression of CC10 in human inferior turbinate [5], we used the human inferior turbinate tissue as a positive control for western blot analysis. As shown in Figure 1, an obvious expression of CC10 protein was found in pCC10 transfected, but not mock transfected, BEAS-2B cells. Immunofluorescence staining revealed CC10 protein expression in the cytoplasm of pCC10 transduced BEAS-2B cells (Figure 1A).

### CC10 inhibits IL-1β induced IL-8 expression in BEAS-2B cells

**Figure 2:**
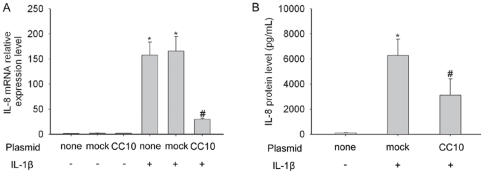
CC10 gene transfer inhibits IL-1β stimulated IL-8 expression in BEAS-2B cells. (A) IL-8 mRNA and (B) protein expression in BEAS-2B cells without transfection, and mock or pCC10 transduced BEAS-2B cells stimulated with or without IL-1β at a concentration of 10 ng/mL. n = 3, ^*^ *P*<0.01 compared with cells without any treatment, ^#^ *P*<0.05 compared with mock transduced cells treated with IL-1β. CC10, Clara cell 10-kDa protein; IL, interleukin.

In order to determine whether induced production of CC10 has an anti-inflammation function in airway epithelial cells, we observed the effect of CC10 gene transfer on IL-1β induced IL-8 expression in BEAS-2B cells. Neither mock nor pCC10 transfer altered the baseline IL-8 mRNA expression levels in BEAS-2B cells (Figure 2A). IL-1β markedly induced IL-8 production in mRNA and protein levels in BEAS-2B cells, which could be significantly attenuated by pCC10 transfer, but not by mock transfer (Figure 2).

### CC10 suppresses NF-κB-mediated transcriptional activity

**Figure 3:**
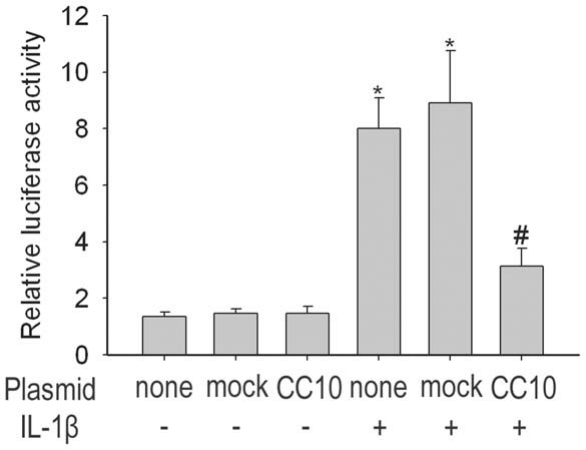
CC10 gene transfer suppresses IL-1β induced NF-κB transcription activity in BEAS-2B cells. BEAS-2B cells transfected with either pCC10 or mock were transiently contransfected with pGL4.32[*luc2P*/NF-κB-RE/Hygro] vector and pGL4.74[hRluc/TK] vector. The firefly luciferase activity was normalized to renilla luciferase activity. n = 3, ^*^ *P*<0.05 compared with cells without any treatment, ^#^ *P*<0.05 compared with mock transfected cells treated with 10 ng/mL of IL-1β. CC10, Clara cell 10-kDa protein; IL, interleukin.

Since IL-1β induced IL-8 expression is mediated through NF-κB signaling pathway, we next detected whether CC10 could affect the activation of NF-κB. First, we assessed whether CC10 could inhibit NF-κB-mediated transcriptional activation. Induction of NF-κB activation in BEAS-2B cells by the inflammatory mediator, IL-1β, was assessed by a luciferase NF-κB reporter construct. BEAS-2B cells had an intrinsic NF-κB activity (Figure 3). Neither mock nor pCC10 transfer altered the baseline NF-κB activity in BEAS-2B cells (Figure 3). IL-1β caused an approximate 6.6-fold increase in relative luciferase activity (Figure 3). Overexpression of CC10 significantly inhibited IL-1β-induced NF-κB activity and the decrease was about 64.7% (Figure 3).

### CC10 prevents NF-κB nuclear translocation

**Figure 4:**
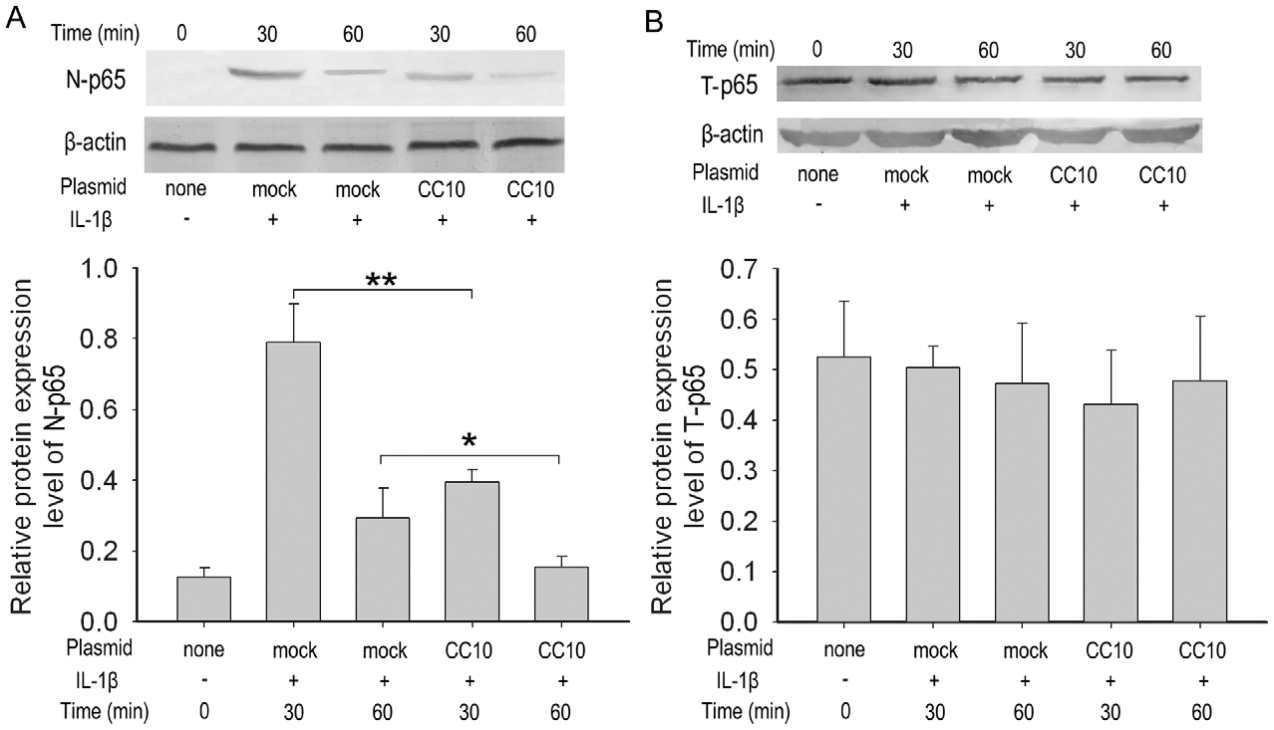
CC10 gene transfer prevents NF-κB nuclear translocation in BEAS-2B cells. BEAS-2B cells transfected with either mock or pCC10 were treated with 10 ng/mL of IL-1β for 30 or 60 minutes. Total and nuclear proteins were extracted and then performed to western blot. (A) Nuclear NF-κB p65 (N-p65) and (B) total p65 (T-p65) were detected. One representative experiment of the three independent experiments is shown. (C) and (D) The semi-quantitative analysis of western-blot gels and results were presented as relative protein expression levels. n = 3, ^**^ *P*<0.01 and ^*^ *P*<0.05. CC10, Clara cell 10-kDa protein; IL, interleukin.

Because activation of NF-κB transcription requires the nuclear translocation of NF-κB, the effect of CC10 on the total and nuclear pool of the NF-κB protein was then assessed by western blot analysis. This study showed that IL-1β treatment effectively increased the nuclear NF-κB p65 protein levels (Figure 4A) but did not change the total NF-κB p65 protein levels (Figure 4B) in mock transduced BEAS-2B cells, which is an indication of the nuclear translocation of NF-κB. CC10 gene transfer did not impact total p65 levels, but significantly suppressed IL-1β induced increase in nuclear p65 levels, suggesting that CC10 could block the NF-κB translocation to cell nucleus (Figure 4).

### CC10 inhibits the phosphorylation of IκB-α, but not IKKα/β

**Figure 5:**
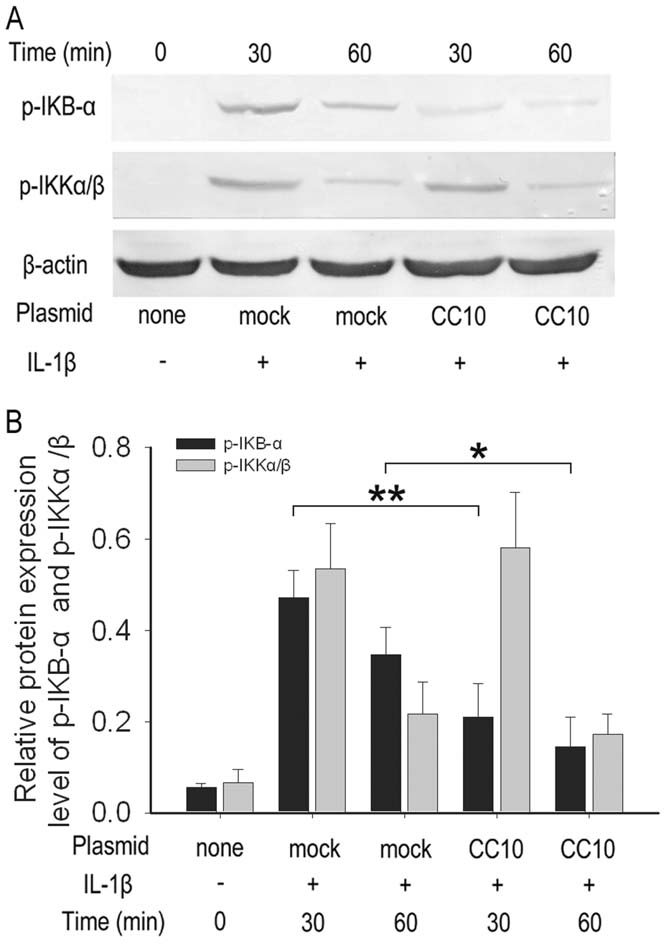
CC10 gene transfer inhibits the phosphorylation of IκB-α but not IKKα/β in BEAS-2B cells. BEAS-2B cells were transfected with either mock or pCC10 and treated with 10 ng/mL of IL-1β for 30 or 60 minutes. (A) Total protein was extracted, and phosphorylated IκB-α and IKKα/β were detected by means of western blot. One representative experiment of the three independent experiments is shown. (B) The semi-quantitative analysis of western-blot gels and results were presented as relative protein expression levels. n = 3, ^**^ *P*<0.01 and ^*^ *P*<0.05. CC10, Clara cell 10-kDa protein; IL, interleukin.

Since phosphorylation of IκB-α is essential for NF-κB degradation, nuclear translocation, and activation in the classic signaling pathway, we then examined the effect of CC10 on the phosphorylation of IκB-α induced by IL-1β via western blotting. As illustrated in Figure 5, IL-1β induced phosphorylation of IκB-α occurred in mock transduced BEAS-2B cells, but was markedly inhibited in pCC10-transduced cells. It is known that phosphorylation of IκB-α is a direct downstream event of phosphorylation of IKKα/β in canonical NF-κB signaling pathway. We therefore examined the CC10's effect on phosphorylation of IKKα/β; however, in this study, we did not find a significant difference in the levels of IKKα/β phosphorylation between mock and pCC10 transfected BEAS-2B cells after IL-1β stimulation (Figure 5).

### CC10 does not interact with NF-κB p65/p50 subunits directly

**Figure 6:**
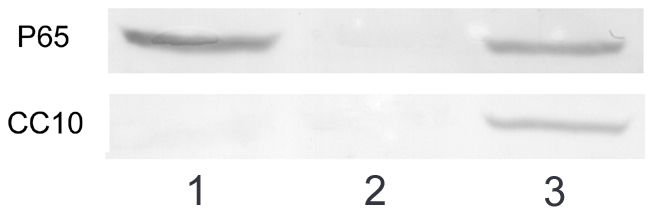
CC10 does not interact with NF-κB p65/p50 subunits directly. BEAS-2B cells transfected with pCC10 were stimulated with 10 ng/mL of IL-1β for 4 hours. Cellular proteins were immunoprecipitated with anti-p65 antibody. Immunoblotting was performed with anti-p65 and anti-CC10 antibodies (lane 1). Rabbit normal serum was served as a negative control for immunoprecipitation (lane 2) and human inferior turbinate mucosa were used as positive controls for CC10 and p65 expression (lane 3). One representative experiment of the three independent experiments is shown.

The direct interaction with NF-κB p65/p50 subunits may affect the nuclear translocation and DNA binding of NF-κB. In this study, we investigated the possible *in vivo* protein-protein interaction between CC10 and NF-κB. Immunoprecipitation of protein extracts from IL-1β stimulated CC10 transfected BEAS-2B cells with anti-p65 antibody followed by western blotting with p65 and CC10 antibodies indicated that CC10 did not coimmunoprecipitate with NF-κB p65/p50 subunits, suggesting that CC10 may not interact with NF-κB p65/p50 subunits directly (Figure 6).

### IL-1β induced IL-8 expression is inversely correlated with CC10 levels in human sinonasal mucosa

**Figure 7:**
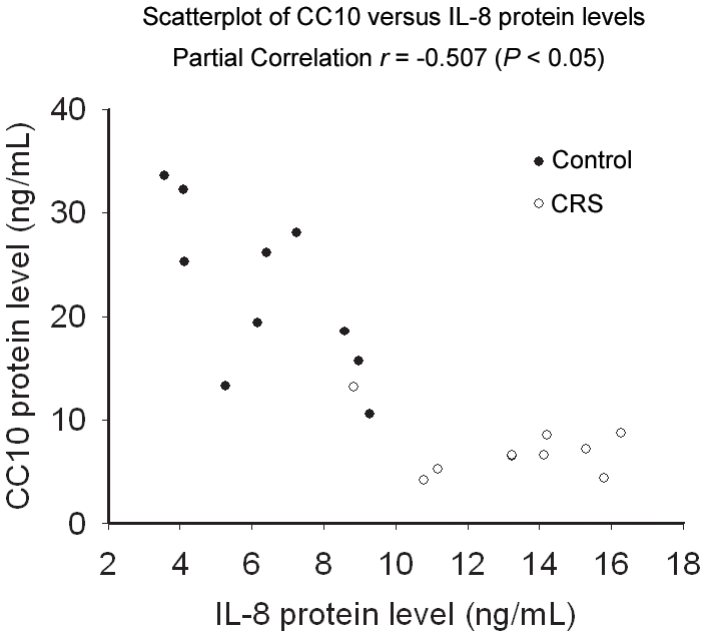
CC10 expression levels are inversely correlated with IL-8 production evoked by IL-1β in human sinonasal mucosa. Sinonasal mucosa obtained from chronic rhinosinusitis and control subjects were cultured *in vitro* and stimulated with 10 ng/mL IL-1β for 24 hours, and then the CC10 levels in tissue homogenates and IL-8 levels in supernatants were determined by means of ELISA. Partial correlations were computed on IL-8 protein and CC10 protein levels after adjustment for diagnostic groups.

Since CC10 could inhibit IL-1β induced IL-8 expression in BEAS-2B cells, we then investigated whether endogenous CC10 expression levels could affect IL-1β induced IL-8 expression in human airway. We found that compared with controls CC10 expression levels was significantly down-regulated in CRS (control vs. CRS, 22.29±7.97 ng/mL vs. 7.12±2.62 ng/mL, *P*<0.01), whereas the IL-1β induced IL-8 expression was significantly enhanced in CRS (control vs. CRS, 6.37±2.11 ng/mL vs. 13.29±2.38 ng/mL, *P*<0.01). Interestingly, the CC10 expression levels were inversely correlated with IL-8 expression levels (Figure 7).

### CC10 gene transfection suppresses IL-1β induced IL-8 expression in nasal epithelial cells in mice

**Figure 8:**
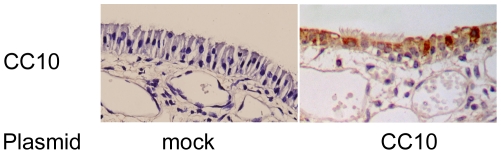
Induction of CC10 protein expression in CC10-knockout mice by gene transfer. Representative photomicrographs of CC10 immunohistochemical staining of sinonasal mucosa sections of mock and pCC10 transfected CC10 knockout mice. CC10 expression was detected three days after transfection. Original magnification ×400.

**Figure 9:**
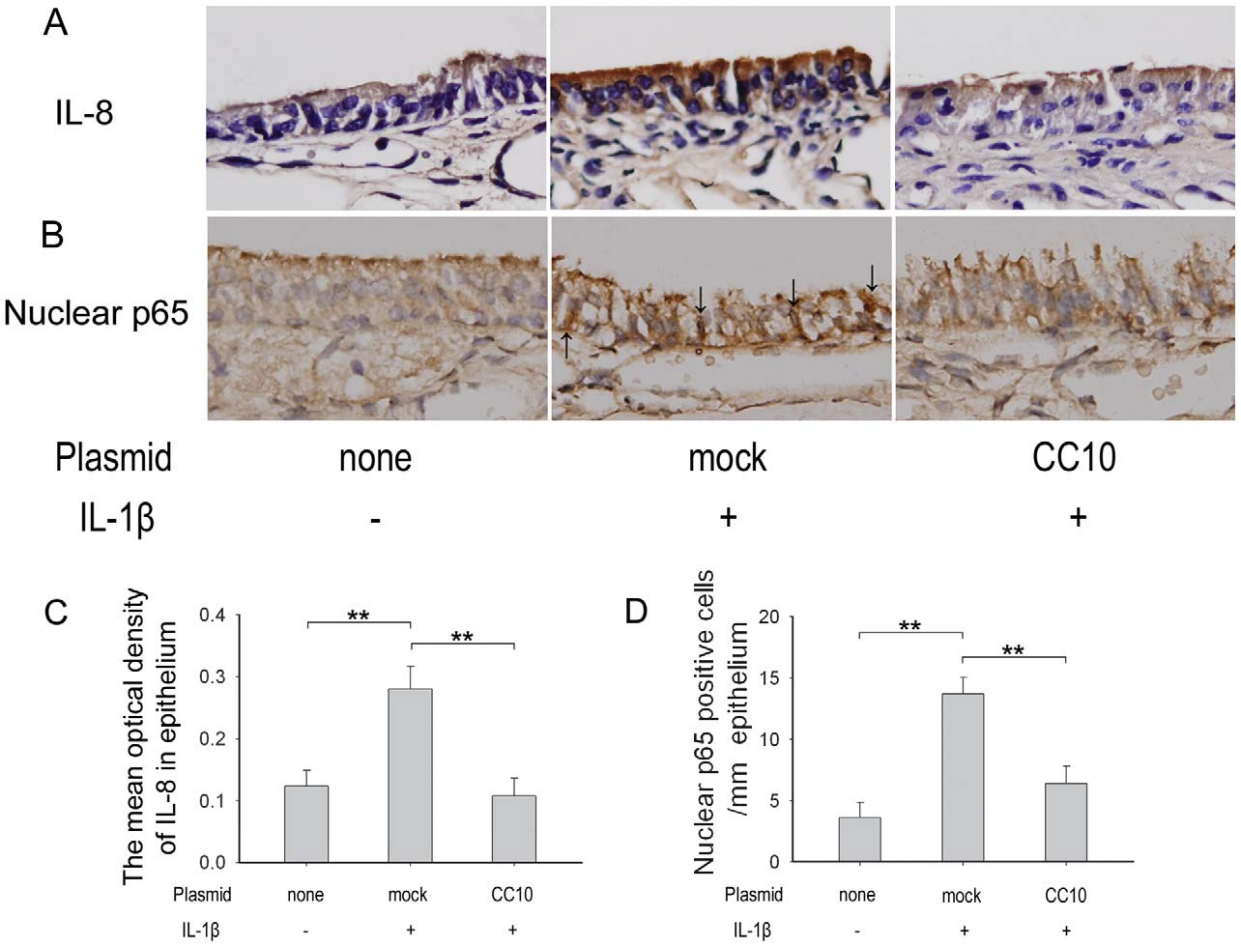
CC10 gene transfer suppresses IL-1β induced IL-8 expression through inhibiting NF-κB nuclear translocation in CC10-knockout mice. (A) and (B) Representative photomicrographs of IL-8 and p65 protein immunohistochemical staining of sinonasal mucosa sections of controls mice without any treatment and mock and pCC10 transfected mice with IL-1β stimulation. Original magnification ×400. *Arrows*, p65 positive staining in nucleus of epithelial cells. (C) The mean optical density of IL-8 in airway epithelium. (D) The number of nuclear p65 positive cells in airway epithelium. n = 6 mice per group ^**^ *P*<0.01.

Finally, we investigated the effect of CC10 transfection on IL-1β induced IL-8 expression *in vivo*. In this study, the induction of CC10 protein expression in nasal epithelial cells in CC10 knockout mice three days after transfection was confirmed by mean of immunohistochemical staining (Figure 8). We found that IL-8 protein was markedly increased in nasal epithelial cells of CC10 knockout mice after local administration of IL-1β, and this increase could be markedly inhibited by pCC10 transfection (Figure 9A and C). Moreover, we found that the nuclear staining of p65 protein was increased after IL-1β administration and pCC10 transfer could attenuate this increase (Figure 9B and D).

## Discussion

In the present paper we found that induction of CC10 expression by gene transfer can inhibit the proinflammatory action of airway epithelial cells *in vitro* and *in vivo*, and described a new mechanism of this effect. In particular, we showed that CC10 gene transfer inhibits NF-κB activity by suppressing the phosphorylation of IκB-α in airway epithelial cells. Given the key role of NF-κB pathway in a lot of biological and pathophysiological processes, our current study underscores the potential therapeutic effect of CC10 gene transfer in various inflammatory diseases.

CC10 is a multifunctional protein with anti-inflammatory and immunomodulatory effects [30]. In the present study, we constructed CC10 plasmid and transfected it to BEAS-2B cells to study the effect of induction of CC10 protein expression on airway inflammation. The human normal bronchial epithelial cell line, BEAS-2B cell, has been validated as a good model for the research of airway epithelial cell function [31]. In this study, we did not find CC10 expression in BEAS-2B cells, which allow us to dissect the influence of induced CC10 expression on the proinflammatory action of airway epithelial cells. After transfection with CC10 plasmid, BEAS-2B cells produced a significant amount of CC10 in cytoplasm. Previous studies have showed that proinflammatory cytokine IL-1β mediated inflammation is one of the central themes in asthma and sinusitis [21]. IL-1β upregulates the expression of a number of proinflammatory genes in airway epithelial cells [21]. Among those, IL-8 is a chemokine attracting neutrophils and also eosinophils [32]. In this study, we studied the modulatory effect of CC10 on IL-1β induced IL-8 expression in BEAS-2B cells. As expected, we found that after IL-1β stimulation, IL-8 mRNA and protein expression was dramatically upregulated in BEAS-2B cells. More importantly, we found that CC10 gene transfer could markedly inhibit such increase, which reveal a potential effect of CC10 gene transfer in controlling airway inflammation.

Activated NF-κB has been considered as a key regulator of inflammation in allergic and inflammatory airway diseases [18], [19]. The evidence of NF-κB activation during airway inflammation has been obtained both in animal models and in human biopsy specimens [33], [34]. NF-κB can be activated by a wide range of stimuli involved in the pathogenesis of allergic and inflammatory airway diseases, such as microbial infection, proinflammatory cytokines, and physical and oxidative stress through so called classical pathway [35]. In classical signaling pathway, NF-κB activity is tightly controlled by the inhibitory protein, IκB-α, which is normally present in the cytosol and complexed to NF-κB dimers, thereby preventing the nuclear localization of NF-κB and ensuring a low basal transcriptional activity. Upon cellular stimulation, IKKα/β, is activated by phosphorylation, which leads to the phosphorylation of IκB-α. The phosphorylated IκB-α is subsequently ubiquitinated and degraded, which exposes the nuclear localization sequence of NF-κB p65/p50 subunits and allows p65/p50 subunits to enter into the nucleus, thus facilitating DNA binding and the transcriptional up-regulation of genes downstream of the κB motif [36], [37]. The activation of NF-κB canonical signaling pathway has been proved to be involved in IL-1β stimulated IL-8 production [38]. Therefore, we investigated whether the inhibitory effect of CC10 on IL-1β induced IL-8 expression is mediated through affecting NF-κB signaling pathway in airway epithelial cells. Indeed, the luciferase reporter assay showed that CC10 gene transfer did suppress the IL-1β evoked NF-κB-mediated transcriptional activity in BEAS-2B cells. Furthermore, we studied the underlying molecular mechanism. We found that CC10 could inhibit nuclear translocation of NF-κB in BEAS-2B cells, which may result from the suppression of phosphorylation of IκB-α. However, no significant effect on the phosphorylation of IKKα/β was found for CC10. Therefore, our present study suggests that CC10 may affect the activation of IκB-α induced by activated IKKα/β, but do not interfere with the activation of IKKα/β. However, how CC10 affects the phosphorylation of IκB-α and whether there is a direct interaction between CC10 and IKKα/β and IκB-α waits for further detailed studies. One limitation of our current study is that we did not investigate the CC10 effect on DNA binding of NF-κB. However, our coimmunoprecipitation experiment revealed that there was no protein-to-protein interaction between CC10 and NF-κB p65/p50 subunits. Therefore, it is less possible that CC10 will affect the DNA binding of NF-κB directly.

We further studied the effect of endogenous CC10 on the regulation of airway inflammation through human nasal explant culture. We found that CC10 expression was down-regulated in human CRS compared with controls, which is consistent with our previous reports [5]. Moreover, we found that IL-1β induced IL-8 expression was enhanced in CRS compared with controls and IL-1β induced IL-8 production was negatively correlated with CC10 expression, further confirming the regulatory role of CC10 in airway inflammation.

Finally, we investigated the effect of CC10 gene transfer on IL-1β induced IL-8 expression *in vivo*. In this study CC10 knockout mice were used in order to avoid potential confounding variables, including variations in the level of endogenous CC10 before and after the acute inflammatory response found in wild type mice [15]. Consistent with the findings in airway epithelial cells, we found that IL-1β could induce the IL-8 expression and the nuclear translocation of NF-κB in nasal epithelial cells in CC10-knockout mice and CC10 plasmid transfection could suppress such change, which further supports the therapeutic function of CC10 gene transfer in controlling airway inflammation. In the present study, although we did not repeat the studies in primary bronchial epithelial cells due to certain technique limitations in gene transfection in primary cells, our *in vivo* experiment clearly confirmed the inhibitory effect of CC10 gene transfection on NF-κB activation in airway epithelial cells.

We have to point out that the other limitation of our current study is that based on the current techniques, it is impossible for us to conclude that the upstream event of CC10's effect on NF-κB suppression is mediated through extracellular or intracellular mechanism. Since CC10 can be secreted out by transfected cells. Until now, despite the great efforts made, the putative surface receptor of CC10 remains enigmatic, which hampers the study of the mechanisms of CC10's action. We believe that more studied are required to address these issues, however, this depends on the technique progress.

In summary, induction of CC10 expression by gene transfer has an inhibitory effect on the IL-1β induced IL-8 expression in airway epithelial cells via blocking NF-κB activity. CC10 inhibits NF-κB activity by suppressing the phosphorylation of IκB-α in airway epithelial cells. These findings may provide a new consideration to treat airway inflammation disease in the future.
